# Robert Bentley Todd, M.D., F.R.S.

**Published:** 1860-05

**Authors:** 


ROBERT BENTLEY TODD, M.D., F.R.S. —It is with feelings of the deepest regret that we are called upon to announce the death of this distinguished member of our profession, which took place on the evening of the 30th of January, 1860, in the fifty-first year of his age. Dr. Todd was the son of the late Mr. Todd, an eminent professor and surgeon in Dublin, who likewise died in the zenith of professional success, and was born on the 9th of April, 1809. He received his medical education at the Richmond Hospital, graduated in Arts at Trinity College, and became a member of the Royal College of Surgeons in 1831. He immediately moved to London, where he was a perfect stranger, and soon became lecturer on anatomy at the Aldersgate Street School; but he afterwards resigned the post for the same position in the Medical School of the Westminster Hospital. In 1835 he began editing his great work, which first brought him into notice as an author, the "Cyclopædia of Anatomy and Physiology," which was completed more than a year since; and a year subsequently he was appointed Professor of Physiology and of General and Morbid Anatomy in King's College. It was due to his untiring efforts that the King's College Hospital was founded, and he held the position of clinical lecturer in this institution up to within a few weeks of his death, being obliged to relinquish the post on account of his failing health. Dr. Todd always stood in the foremost rank as a physiological investigator, his conclusions being based upon an accurate knowledge of minute structural anatomy; and in 1845, in conjunction with Mr. Bowman, he published the first part of the "Physiological Anatomy and Physiology of Man." After fourteen years of incessant labor the work was completed, having appeared in successive parts, and immediately became the standard authority upon the various subjects treated of. The chapter on the nervous system tended more to give a shape to the affections of this portion of the organism than any other publication previous to the time of its appearance.

As physician to the Western Dispensary and the Royal Infirmary for Children, as well as lecturer at King's College Hospital, Dr. Todd had an ample field for clinical study and teaching, and we need only point to his "Clinical Lectures" to show how good a use he made of his opportunities, not neglecting the study of medicine, while arduously pursuing his physiological investigations. As a clinical teacher, he was clear, forcible, and practical; he was also an accurate diagnostician, and he effected great changes in the ordinary routine practice of medicine.

Dr. Todd was a voluminous author; and taking his works in the order of their appearance or completion, we find that he published in 1843, "Remarks on Gout, Rheumatic Fever, and Chronic Rheumatism of the Joints;" in 1845, the "Descriptive and Physiological Anatomy of the Brain, Spinal Cord, and Ganglions, and of their Coverings;" in 1856, "Clinical Lectures on Paralysis, certain Diseases of the Brain, and other Affections of the Nervous System;" in 1857, Clinical Lectures on certain Diseases of the Urinary Organs, and on Dropsies, and, in connection with Mr. Bowman, the completion of the "Physiological Anatomy, and Physiology of Man;" in 1859, the "Cyclopædia of Anatomy and Physiology;" and finally, in 1860, shortly before his death, "Clinical Lectures on certain Acute Diseases."

The reputation of Dr. Todd will be perpetuated more particularly by his clinical lectures, or, to use the words of the *Lancet*, from which we have gleaned some facts contained in this notice, "Dr. Todd regarded none of his discoveries with more satisfaction than his establishment of the fact of the co-ordinating power of the posterior columns of the spinal cord; and this piece of pure inductive reasoning he had the gratification of finding confirmed of late years by the elaborate experiments of Dr. Brown-Séquard."

Owing to his laborious practice, Dr. Todd was obliged to resign his Chair of Physiology in 1853, and in December last withdrew from the hospital, of which he had so long been a shining ornament. His death was very sudden; having been called to Wales, on his return he felt ill, but thought he could prescribe for a few urgent cases. At two o'clock, on the thirtieth of January, he was taken in his consulting room with hemorrhage from the stomach, which recurred so severely as to occasion his death early in the evening. The friends of Dr. Todd, and the staff of the King's College Hospital, have determined to erect a statue to his memory, and to found a Todd Clinical Gold Medal as a prize.

